# Climate change, snow mold and the *Bromus tectorum* invasion: mixed evidence for release from cold weather pathogens

**DOI:** 10.1093/aobpla/plz043

**Published:** 2019-07-16

**Authors:** Danielle M Smull, Nicole Pendleton, Andrew R Kleinhesselink, Peter B Adler

**Affiliations:** 1 Department of Wildland Resources and the Ecology Center, Utah State University, Logan, UT, USA; 2 Department of Ecology and Evolutionary Biology, University of California, Los Angeles, Los Angeles, CA, USA

**Keywords:** Cheatgrass, plant disease, sagebrush steppe, sage-grouse, snow mold, subnivean pathogens

## Abstract

Climate change is reducing the depth and duration of winter snowpack, leading to dramatic changes in the soil environment with potentially important ecological consequences. Previous experiments in the Intermountain West of North America indicated that loss of snowpack increases survival and population growth rates of the invasive annual grass *Bromus tectorum*; however, the underlying mechanism is unknown. We hypothesized that reduced snowpack might promote *B. tectorum* population growth by decreasing damage from snow molds, a group of subnivean fungal pathogens. To test this hypothesis, we conducted greenhouse and field experiments to investigate the interaction between early snowmelt and either fungicide addition or snow mold infection of *B. tectorum*. The greenhouse experiment confirmed that the snow mold *Microdochium nivale* can cause mortality of *B. tectorum* seedlings. In the field experiment, early snowmelt and fungicide application both increased *B. tectorum* survival, but their effects did not interact, and snow mold inoculation had no effect on survival. We did find interactive effects of snowmelt and fungal treatments on *B. tectorum* seed production: with ambient snowpack, *M. nivale* inoculation reduced seed production and fungicide increased it, whereas in the early snowmelt treatment seed production was high regardless of fungal treatment. However, treatment effects on seed production did not translate directly to overall population growth, which did not respond to the snow melt by fungal treatment interaction. Based on our mixed results, the hypothesis that reduced snowpack may increase *B. tectorum* fitness by limiting the effects of plant pathogens deserves further investigation.

## Teaser

Climate change and biological invasions are two primary drivers of global change. They can also have interactive effects. We studied the potential for climate change to promote the growth of a destructive invasive annual grass, *Bromus tectorum*, by reducing snow pack and releasing the plant from snow mold infections. We found evidence that snow molds do limit seed production but not survival or overall population growth. The potential for climate change to reduce the impact of cold weather pathogens deserves further investigation.

## Introduction

In many temperate zones, warming associated with climate change is reducing the depth and duration of seasonal snowpacks (Knowles *et al.* 2006; Clow 2009; Stewart 2009) leading to dramatic changes in the soil environment. Thick snowpacks insulate the soil from fluctuations in air temperature (Cline 1997), while soils under thin snowpacks experience wider temperature fluctuations, more frequent freeze-thaw cycles and freezing to greater depths. These changes in soil temperature regimes can alter nutrient cycling and plant community composition (Harte and Shaw 1995; Brooks and Williams 1999; Groffman *et al.* 2001; Wahren *et al.* 2005; Monson *et al.* 2006; Wipf *et al.* 2006).

Most of our knowledge about the ecological consequences of reduced snowpacks comes from arctic and alpine ecosystems (Kreyling 2010), but the effects could be even stronger in warmer and drier ecosystems with shallower snowpacks (Henry 2008) where the duration of snowpack is expected to decline most dramatically (Mastin *et al.* 2010). Typically, deep alpine snowpacks require large reductions in depth before falling below the 30–40 cm insulation threshold, while in a grassland or shrubland, slight reductions in already shallow snowpacks would have an immediate effect on soil temperature and moisture dynamics. In ecosystems characterized by summer drought, most processes slow significantly in summer (e.g. Burke 1989), and biogeochemical cycling occurs during winter and snowmelt. In addition, much of the total plant biomass in these ecosystems occurs below ground in the shallow soil layers that will be subjected to more intense temperature fluctuations and freeze-thaw cycles with reduced snowpacks (Hooker *et al.* 2008). Despite the potential for profound effects, a recent review highlighted a lack of winter climate change studies in temperate steppe and shrubland ecosystems (Kreyling 2010).

A second reason that temperate grasslands and shrublands are of special concern is that they have undergone dramatic shifts in plant species composition over the last century, often involving the replacement of native perennial grasses by woody (Miller and Wigand 1994; van Auken 2000) or invasive species (Mack 1981). These threshold dynamics could be promoted by reductions in snowpack and accompanying alterations in freeze-thaw cycles (Inouye 2008), changes in nutrient cycling (D’Antonio and Vitousek 1992) or feedbacks between vegetation and snow accumulation, ablation rates and soil moisture recharge (Bradford and Lauenroth 2006; Schlaepfer *et al.* 2012a, b).

Reduced snowpack could also impact plant species composition by altering plant–pathogen interactions. Recent reviews of crop and natural systems have argued that interactions between climate change and pathogens have great potential to impact plants, but that more research is needed to reduce the uncertainty about these effects (Tylianakis *et al.* 2008; van der Putten *et al.* 2010; Luck *et al.* 2011; Pautasso *et al.* 2012). Furthermore, most existing work focuses on pathogens active during the growing season, not on the few pathogens capable of infecting plants in winter, such as snow molds. We currently do not know whether climate change, and dramatic reductions in snowpack in particular, will interact with such mechanisms to exacerbate vegetation shifts and plant invasions in semi-arid ecosystems.

*Bromus tectorum* (cheatgrass), the most widespread exotic annual grass in North America, has degraded tens of millions of hectares of the sagebrush steppe (Bradley and Mustard 2006). *Bromus tectorum* creates a continuous layer of fine fuels that dramatically decreases fire return intervals (Bukowski and Baker 2012; Balch *et al.* 2013). The increased fire frequency has negatively impacted native plant communities and their obligate consumers, including the Greater Sage-grouse, a species of conservation concern (Knick *et al.* 2003; Hanser and Manier 2013). The invasion of *B. tectorum* has also decreased the value of rangelands within the Great Basin. *Bromus tectorum* loses its palatability and nutritional value faster than native perennial bunchgrasses (Tisdale and McLean 1954); it is also a less reliable forage source, particularly during drought years (Stewart and Hull 1949).

Anticipating the trajectory of the *B. tectorum* invasion under climate change is a high priority given the threat it poses to sagebrush ecosystems, and the massive current investments in sagebrush conservation and restoration (Department of Interior Secretarial Order 3336 in 2015). Currently, *B. tectorum* impacts are greatest in the hot, dry, low elevation valleys of the Great Basin, while sagebrush habitat is relatively intact in cooler, moister areas at higher elevations and east of the Continental Divide (Chambers *et al.* 2014a, b). However, experiments (Chambers *et al.* 2007; Concilio *et al.* 2013; Compagnoni and Adler 2014b; Blumenthal *et al.* 2016) and species distribution models (Bradley 2009; Boyte *et al.* 2016) suggest that warming could increase *B. tectorum* impacts in the higher elevation, cooler portions of the range that have so far shown more resistance and resilience to invasion. Reduced snowpack could provide a mechanism to explain why the fitness of *B. tectorum* at higher elevations might increase under a warmer climate.

A recent warming experiment showed that decreased duration of winter snowpack benefits *B. tectorum* by increasing overwinter seedling survival (Compagnoni and Adler 2014a). However, exactly why reduced snowpack increased *B. tectorum*’s performance remains unclear. Warming may directly benefit *B. tectorum* by advancing the onset of the spring growing season and freeing vulnerable seedlings from carbohydrate depletion. Another possible benefit of reduced snowpack is escape from infection of fungal pathogens such as snow molds.

Snow mold is a generic term for fungi that grow beneath snow and, if pathogenic, infect plants over winter. The most common of these in North America is *Microdochium nivale*, also known as Pink Snow Mold. A frequent pathogen of winter wheat, *M. nivale* is also anecdotally reported to infect *B. tectorum* in the USA (Sprague 1953; Klemmedson and Smith 1964). However, the impact of this infection on *B. tectorum* demography and population growth remains unknown.

Under persistent snowpack, *M. nivale* could cause overwinter mortality of *B. tectorum* seedlings, as with winter wheat. Furthermore, seedlings that escape mortality may still suffer foliar damage or stunted growth due to infection and produce less seed at maturity, as is documented in some other fungal infections of plants (Modjo and Hendrix 1986). Research on winter wheat has shown that mortality due to *M. nivale* decreases as the duration of snow cover decreases (Nakajima and Abe 1994). As climate change causes more winter precipitation to fall as rain and snowpack duration declines, the potential for *M. nivale* to reach the abundances necessary to infect and kill grass seedlings may also decline. Ultimately, this could increase *B. tectorum*’s survival, seed production and population growth rate in parts of its range which historically had persistent winter snowpack.

While *M. nivale* is not the only pathogen of *B. tectorum* that might respond to climate change (Meyer *et al.* 2016), it is uniquely sensitive to loss of snow, a certain consequence of warming. Prevéy and Seastedt (2015) found that infection of *B. tectorum* by head smut (*Ustilago bullata*) was promoted by wet winter conditions in a site in eastern Colorado, where winters tend to be dry. In contrast, the seed pathogens *Pyrenophora semeniperda* and *Fusarium* infect and kill higher fractions of *B. tectorum* seeds in drier sites (Beckstead *et al.* 2007; Meyer *et al.* 2007, 2008, 2014). Changes in precipitation regimes could increase or decrease the impacts of these pathogens on *B. tectorum*. However, future changes in precipitation amount are uncertain. In contrast, regional climate models are confident in projecting reduced snowpack duration across the range of *B. tectorum* (Mastin *et al.* 2010), which will directly limit the influence of snow molds.

Here, we report on (i) a greenhouse experiment to confirm that *M. nivale* infects *B. tectorum* seedlings and can cause mortality, and (ii) a split-plot field experiment to examine interactions between snowpack duration and the effects of *M. nivale* on *B. tectorum.* In the field experiment, we used infrared lamps to induce early snowmelt of plots planted with *B. tectorum* seeds and augmented or reduced *M. nivale* using inoculate or fungicide, respectively. To support our hypothesis that snow mold can limit *B. tectorum* fitness, we needed evidence for a statistical interaction between the snowmelt and fungicide treatments: Under intact snowpack, the addition of snow mold cultures should reduce *B. tectorum* performance at the individual (emergence, survival, fecundity) or population level while fungicide would increase performance relative to controls, but in the absence of persistent snowpack, neither snow mold nor fungicide should impact individual vital rates or population growth.

## Materials and Methods

### Greenhouse experiment

The purpose of our greenhouse experiment was to determine the potential for seedling damage and mortality due to *M. nivale* under snow-like conditions. We focus on the seedling stage because it should be most vulnerable to infection, and is also the stage in which fall-germinating *B. tectorum* plants persist through winter. We began by collecting samples of *M. nivale* in Logan, UT, USA, and culturing the samples on potato-dextrose agar with streptomycin at 12 °C for 3 weeks. We then isolated a single strain of *M. nivale*, identified via its cone-shaped conidia, pink colour and ability to grow at low temperature. From this pure culture, we transferred mycelial plugs to a sterilized, hydrated mixture of oat, barley and cracked corn. Autoclave sterilization killed the grain; there was no germination in either the greenhouse or field experiment. The inoculated grain was incubated in a dark growth chamber at 10 °C for 3 weeks.

We then raised *B. tectorum* seedlings to inoculate with our cultures of *M. nivale*. Seeds collected in Logan, UT, were sterilized in a low concentration bleach solution and germinated on moist blotter paper after 3 days of 12-h cycles of 20 °C/light and 10 °C/dark. We then transplanted the germinated seeds in 33 g of soil in conical vials. Soils were previously steam-sterilized in an autoclave. *Bromus tectorum* seedlings were raised in ambient greenhouse conditions and were watered daily. After most seedlings had two true leaves (~2 weeks), we cold-hardened them in a 4 °C lit growth chamber for 10 days.

After the cold-hardening period, we randomly assigned each seedling to receive one of two grain treatments: sterile grain (the control) or grain inoculated with *M. nivale*. We assigned 126 plants to each treatment. The cones were housed in three trays (blocks), with the location of plants randomized within each tray. To prevent cross-contamination between plants, we placed transparent plastic tubes around each conical vial containing a seedling. We then added 5 g of the assigned grain to each seedling. Since we could not maintain snow in the growth chamber, we placed sterile cotton balls moistened with water into each tube to simulate the moist conditions seedlings would face under snowpack. Seedlings were then incubated in dark growth chambers at 2 °C for 3 weeks. After this winterized period, cotton balls were removed and the plants were returned to ambient greenhouse conditions after a period of acclimatization. After 3 weeks of recovery time, we measured mortality of seedlings.

### Field experiment

Our field experiment was conducted at Green Canyon ecological station, near the Utah State University campus in Logan, UT, USA, ~1460 m above sea level. Vegetation at the experiment site is dominated by *Artemisia tridentata* and *Pseudoeroegneria spicata*. *Bromus tectorum* is present in low densities (personal observation).

We installed a split-plot experiment in September 2015. The whole-plot treatments, control and early snowmelt, were each replicated six times. Early snowmelt treatments were implemented by melting snow using infrared heat lamps (Model HS-2420, Kalgo Electronics Company) mounted 1.2 m above the ground. On 9 January 2016, following a full month of snowpack at the study site, the lamps were turned on until the 50 cm of snow within the snowmelt treatment plots had melted (24 h). Interannual variation in snowpack at this site is high and mid-winter thaws are not uncommon. We began the snowmelt treatment in early January because we could not be sure we would have snow to melt in February. We repeated this process to melt snow from each subsequent snowfall event until snowpack in the control plots had melted (8 March). Our previous experiment showed negligible effects of the snowmelt treatment on soil moisture and soil temperature (Compagnoni and Adler 2014b).

Within each plot, we installed four subplots, each consisting of 25 × 25 cm square plastic mesh grids that contained 100 2.5 × 2.5 cm cells. Each subplot was cleared of vegetation to minimize the effect of interspecific competition from perennial grasses. In September, we planted three of these grids with locally collected *B. tectorum* seed; a single seed was planted in each cell of the grid, for a total of 100 potential plants per subplot. The resulting density of the three planted grids was 1600 seeds per m^2^, much lower than high-density monoculture stands where densities may exceed 15 000 seeds per m^2^ (Stewart and Hull 1949). This allowed us to assess the impact of warming and snow mold on *B. tectorum* in the absence of strong intraspecific competition. The fourth quadrat was left unplanted and used to assess background *B. tectorum* seedling emergence. We found that background emergence was negligible, and therefore did not need to correct for it in subsequent analyses.

The three planted subplots were randomly assigned to one of the split-plot fungal manipulation treatments: control, snow mold addition or fungicide addition **[see****Supporting Information—Fig. S1****]**. Control subplots did not have their fungal communities manipulated. Fungicide subplots were sprayed with a commercial fungicide designed to target snow molds, including *M. nivale*. Each quadrat was sprayed with a 0.31 % Azoxystrobin solution (Heritage® G) in late October according to manufacturer’s instructions. Azoxystrobin is a commercial fungicide approved to target *M. nivale* and other snow molds on turfgrasses.

For the snow mold treatment, fungal levels were augmented using a wheat seed culture of *M. nivale* prepared using the same methods as in the greenhouse experiment described above. We spread 200 mL of the inoculated wheat seed on each of the snow mold subplots in late November, prior to the formation of winter snowpack. Control and fungicide subplots were sowed with sterile wheat seed. Hardware mesh cages were installed over all subplots to deter herbivory by small mammals.

We monitored seedling emergence, survival, biomass production, seed production and population growth rates. Distinguishing between the effects of snow mold on individual performance measures (emergence, survival, biomass and seed production) can provide insight into the mechanisms by which the pathogens impact the host. Population growth rate integrates across survival and seed production to provide a measure of overall fitness. To estimate vital rates, we censused survival of each individual seedling in each subplot on a biweekly basis from March until May. We harvested all above-ground biomass after mature *B. tectorum* plants had set seed, in early July. We determined the final above-ground biomass of surviving *B. tectorum*, the above-ground biomass of non-*B. tectorum* vegetation and the *B. tectorum* seed production of each subplot. We also counted the number of spikelets infected by the head smut *U. bullata*, identified by the presence of black, soot-like spores instead of seeds, to determine if our treatments affected other fungal pathogens of *B. tectorum*.

We calculated the geometric population growth rate (λ) in each subplot as λ = *n*_*t*+1_/*n*_*t*_ where *n*_*t*_ is the number of seeds at time *t*. *n*_*t*_ was equal to 100, as 100 seeds were planted in each subplot and emergence from the seed bank in unseeded subplots was negligible. *n*_*t*+1_ is the subplot’s total observed seed production. This calculation assumes that planted seeds that fail to germinate in year *t* do not survive and contribute to the seed bank in year *t* + 1; previous studies support this assumption, as *B. tectorum* seeds are short-lived in the seed bank (Smith *et al.* 2008; Compagnoni and Adler 2014b).

#### Analyses

For our greenhouse experiment, we analysed survival of seedlings using a generalized linear mixed model with a binomial distribution, with snow mold addition as a fixed effect and seedling tray as a random effect. To analyse results of the field experiment, we modelled emergence of seedlings and seedling survival (calculated across the full growing season) using a generalized linear mixed model with a binomial distribution. Per capita seed production and number of spikelets per plants infected by head smut were both estimated using generalized linear mixed models with a negative binomial distribution. We log-transformed λ values and modelled the geometric population growth rate using a linear mixed model. Per capita biomass was also modelled with a linear mixed model. For all of these models, temperature treatment and fungal treatment were both fixed effects, and plot was a random effect.

We relied on likelihood ratio tests for hypothesis testing, using the ‘anova’ function in base R. First, we compared the full model, with snowmelt and fungal treatment interactions, against a simpler model including only the main effects of snowmelt and fungal treatment. This is the most important test, because evidence for snow mold impact requires an interaction between the snowmelt and fungal treatments. Next, we compared the model with both main effects to a simpler model which included only the snowmelt effect, dropping the main effect of fungal treatments. By testing the fungal treatments in the presence of the snowmelt effect, we provide a strict test of the fungal treatments. We performed this test, rather than a test of the snowmelt treatment in the presence of the fungal effects, because the fungal treatments are the novel aspect of the experiment (the main effect of snowmelt was demonstrated in a previous experiment). Finally, we compared the snowmelt main effect model to a null model which included no main effects. We used the emmeans package for *post hoc* comparisons of individual treatment effects, adjusting *P*-values for multiple comparison based on Tukey’s method and using the Kenward–Roger method to estimate degrees of freedom.

We conducted all analyses in R version 3.4.1 (R Development Core Team 2017) using the lme4 package for binomial responses (Bates *et al.* 2015) and the glmmADMB package for negative binomial responses (Fournier *et al.* 2012). We made statistical tables using the texreg package (Leifeld 2013).

## Results

### Greenhouse experiment

**Figure 1.**
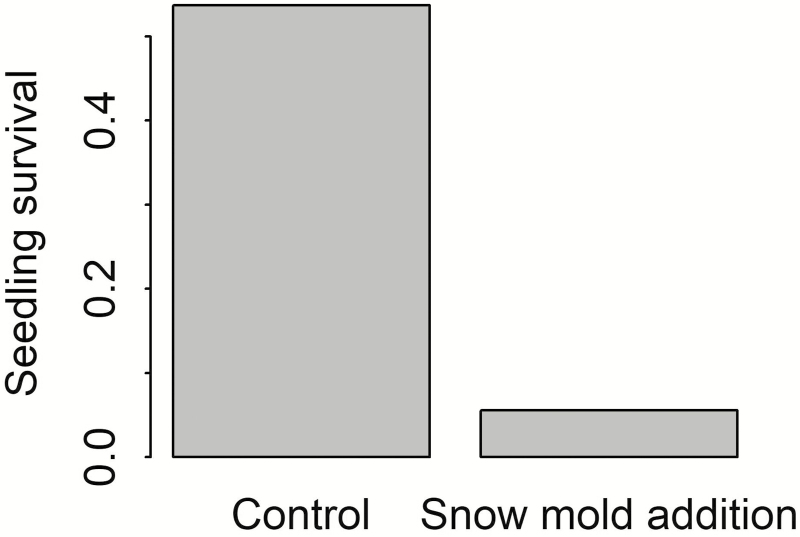
The proportion of seedlings surviving at the end of the greenhouse experiment. Number of plants for each treatment was 121 and 126, respectively.

In the greenhouse experiment, seedling survival was significantly higher for the control treatment (54 %) than for the snow mold inoculation treatment (5.5 %) (**see****Supporting Information—Table S1**, *z* = −6.94, *P* < 0.001; Fig. 1).

### Field experiment

In our field experiment, the emergence rate of *B. tectorum* did not vary significantly with fungicide addition, snow mold addition or snowmelt treatments **[see****Supporting Information—Table S2**, **Fig. S2A****]**. Even when we dropped the snowmelt × fungal treatment interactions, the main effects were not significant. Overall, emergence rates of seedlings were high (78.8 %).

**Figure 2.**
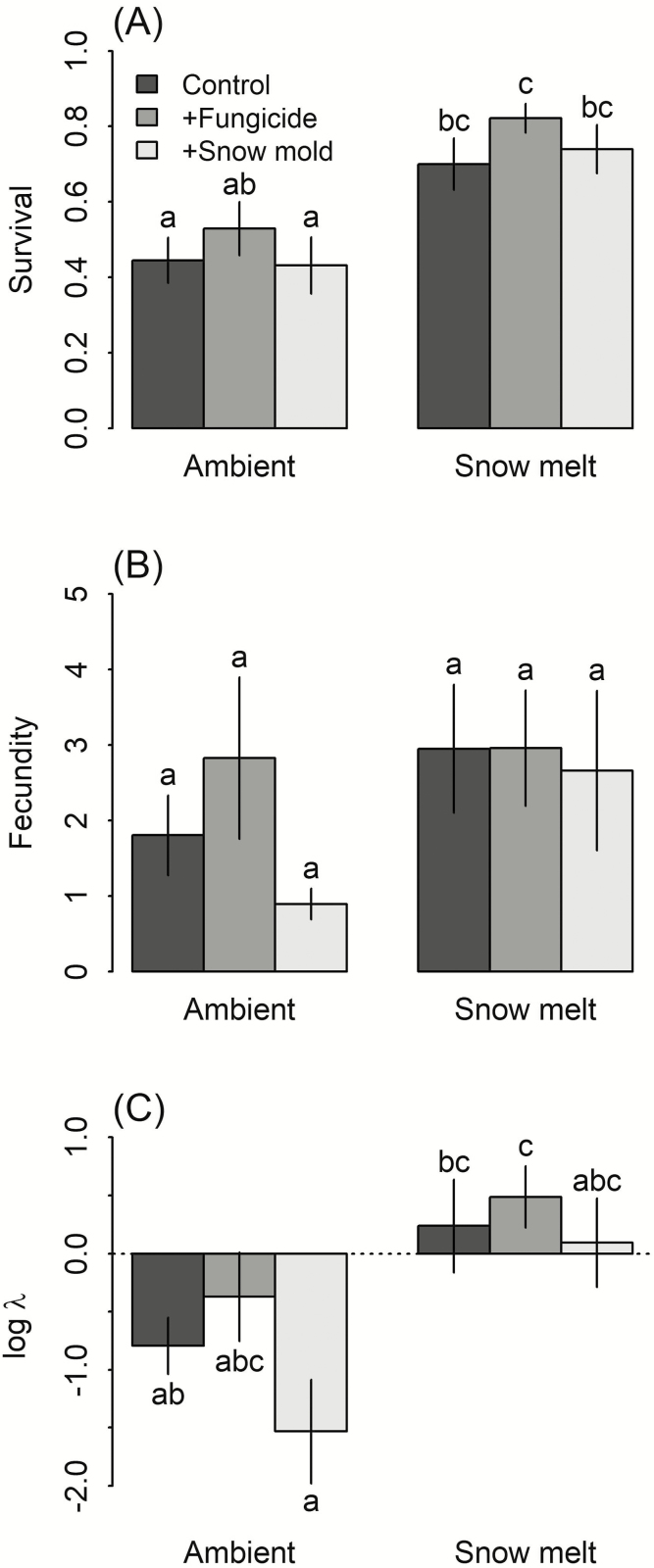
Survival (A), fecundity (B), and population growth rates (C) observed during the field experiment. Values represent treatment means and error bars show ±1 SE. Letters indicate statistically significant differences between treatments based on custom contrasts. Note that fecundity treatment means (B) were estimated using a negative binomial model with a log-link; values shown here were back-transformed.

Seedling survival was significantly higher in plots receiving early snowmelt (Table 1; Fig. 2A). While only 53 % of seedlings that germinated in the ambient plots survived the winter, 82 % of seedlings in the early snowmelt plots survived the winter. Most mortality occurred over winter: of the plants alive in early March, 93 % were still alive at the time of the July harvest. Results of a likelihood ratio test indicated that the interaction terms should be dropped but both main treatment effects retained (Table 1). In the main effects-only model, fungicide had a significant positive effect on survival **[see****Supporting Information—Table S3****]**.

**Table 1.** Likelihood ratio tests for survival models. Each row, except for the first, compares the listed model with the simpler model in the previous row. The fungal main effect includes both a fungicide treatment and a snow mold treatment.

Model	AIC	LogLik	Deviance	χ ^2^	df	*P*-value
NULL	3252.47	−1623.23	3246.47			
Snowmelt	3243.55	−1617.77	3235.55	10.92	1	0.0010
Snowmelt + fungal	3241.52	−1614.76	3229.52	6.03	2	0.0490
Snowmelt × fungal	3244.77	−1614.39	3228.77	0.74	2	0.6901

We found evidence for significant interactive effects of snowmelt and fungal treatments on per capita seed production (Table 2; **Supporting Information—Table S4**; Fig. 2B). Under ambient conditions snow mold addition reduced seed production in comparison with control subplots, while the addition of fungicide increased seed production relative to controls. In contrast, with early snowmelt, there was little difference in seed production between control, snow mold addition and fungicide addition subplots. However, the significant likelihood ratio test for the interaction term did not translate into significant pairwise differences between individual treatments after adjusting for multiple comparisons (Fig. 2B).

**Table 2.** Likelihood ratio tests for fecundity models.

Model	AIC	LogLik	χ ^2^	df	*P*-value
NULL	390.51	−192.25			
Snowmelt	390.74	−191.37	1.77	1	0.1839
Snowmelt + fungal	392.04	−190.02	2.70	2	0.2587
Snowmelt × fungal	388.89	−186.45	7.15	2	0.0280

Treatment effects on biomass production were also weak **[see Supporting Information—Tables S4****and****S5**, **Fig. S2B****]**, but the trends followed a similar pattern as seed production, with a marginally significant snowmelt × fungal treatment interaction (*P* = 0.080). There was a significant linear relationship between subplot *B. tectorum* biomass and subplot seed production (*t* = 19.302, df = 34, *P* < 0.0001; **see****Supporting Information—Fig. S3**), suggesting that the treatments could influence seed production through their effects on biomass.

We found no interactive effects of snowmelt and fungal treatments on the rate of infection by head smut (*U. bullata*), but the main effect of the fungal treatments was significant (Table 3; **see****Supporting Information—Table S4**). Snow mold inoculation was associated with increased infection rates while fungicide application led to lower infection rates, though *post hoc* contrasts showed no significant differences in infection among treatments **[see****Supporting Information—Fig. S2C****]**.

**Table 3.** Likelihood ratio tests for models of head smut infection.

Model	AIC	LogLik	χ ^2^	df	*P*-value
NULL	363.21	−178.61			
Snowmelt	364.60	−178.30	0.62	1	0.4318
Snowmelt + fungal	360.74	−174.37	7.85	2	0.0197
Snowmelt × fungal	363.55	−173.78	1.19	2	0.5505

Geometric population growth rates (λ), analysed on the log scale, were significantly affected by both the snowmelt and fungal treatments, though the snowmelt × fungal treatment interaction was not significant (Table 4; **Supporting Information—Table S4**; Fig. 2C). Growth rates were higher under early snowmelt conditions than ambient snowpack. Compared to the control subplots, population growth was higher under the fungicide treatment and similar or lower in the snow mold treatment. The mean (log) population growth rate under ambient conditions was less than zero, indicating population decline, while mean population geometric growth rates under early snowmelt conditions were positive (Fig. 2C).

**Table 4.** Likelihood ratio tests for models of the population growth rate, λ.

Model	AIC	LogLik	Deviance	χ ^2^	df	*P*-value
NULL	103.69	−48.84	97.69			
Snowmelt	98.46	−45.23	90.46	7.23	1	0.0072
Snowmelt + fungal	94.68	−41.34	82.68	7.77	2	0.0205
Snowmelt × fungal	96.10	−40.05	80.10	2.58	2	0.2747

## Discussion

Recent syntheses have concluded that climate change is likely to interact with plant pathogens in both crop systems (Luck *et al.* 2011) and natural communities (Tylianakis *et al.* 2008; Pautasso *et al.* 2012), with both positive and negative effects possible. In temperate zones, one of the most dramatic impacts of climate change will be the reduction in the depth and duration of snowpacks. Because snowpacks profoundly influence the soil micro-environment, their loss has the potential to drive strong ecological responses. In a previous experiment, we found that loss of snowpack increased the fitness of *B. tectorum* (Compagnoni and Adler 2014a), a destructive invasive annual in western North America. The purpose of the current study was to test the hypothesis that snow mold was the mechanism limiting *B. tectorum* vital rates and overall fitness under snow, and that loss of snow might release the plant from infection.

We found mixed evidence for this hypothesis. A number of our findings show the potential for snow mold to limit *B. tectorum* fitness. Our greenhouse experiment demonstrated that addition of *M. nivale* significantly decreased survival of *B. tectorum* seedlings under simulated snowpack conditions (Fig. 1), confirming previous anecdotal accounts of *B. tectorum* infection by *M. nivale* (Sprague 1953; Klemmedson and Smith 1964). Furthermore, in the field experiment we found a significant interaction (Table 2) between the effect of snow mold and the effect of early snowmelt on seed production, consistent with our hypothesis. Under ambient snowpack, addition of snow mold decreased per capita seed production and addition of fungicide significantly increased per capita seed production (Fig. 2B). In contrast, in the early snowmelt treatment, seed production was similar in control, snow mold and fungicide addition subplots. These results suggest that snow mold may play a role in driving the positive response of *B. tectorum* to the early snowmelt treatment.

On the other hand, we did not find clear evidence for interactive effects of the snowmelt and fungicide treatments on seedling survival in the field experiment. While the early snowmelt treatment significantly increased seedling survival rates over control plot rates during winter (Fig. 2A), consistent with the findings of Compagnoni and Adler (2014a), the snow mold addition did not increase seedling mortality (Fig. 2A), and fungicide addition increased seedling survival in both ambient snowpack and early snow melt treatment. Both results are inconsistent with our hypothesis: if snow mold were the primary fungal pathogen, we would expect a stronger effect of snow mold inoculation on seedling mortality in the ambient snow treatment and a weaker effect of fungicide in the snow melt treatment. In other words, evidence for snow mold as the mechanism for lower survival of *B. tectorum* in the ambient snowpack treatment was weak.

The impact of our treatments on *B. tectorum* fitness requires integrating across individual survival and seed production by calculating the geometric population growth rate. We found that early snowmelt caused substantial increases in population growth rates, consistent with Compagnoni and Adler (2014b). Fungal treatment also significantly affected the population growth rate (Fig. 2C). However, the lack of a significant snowmelt by fungal treatment interaction means we cannot attribute the changes in population growth rate to snow mold infection.

Overall, our results provide a strong indication that *B. tectorum* populations at mid-elevational sites may increase as climate change reduces snowpack depth and duration. We are less confident about the underlying mechanism. The seed production results suggest that a release from snow mold infection may play a role, while the lack of an interactive effect of snowmelt and fungal treatments on survival and population growth rate do not support that hypothesis. Alternative mechanisms might include the effects of reduced snowpack on growing season length or induced dormancy.

A thorough evaluation of the potential for a release from snow mold to promote *B. tectorum* would need to characterize the distribution of snow molds across broad climate and elevation gradients. Furthermore, future work should consider the effects of snowpack and snow molds on *B. tectorum*’s native grass competitors. If the natives benefit more than *B. tectorum* from future loss of snowpack, they could potentially keep the invader in check. However, the perennial native populations appear less sensitive to pathogens which primarily affect seeds and seedlings (Mordecai 2013), and the native grass community is most resistant and resilient to invasion in cool, moist portions of the range (Chambers *et al*. 2014a, b) with greater depth and duration of snowpack. We have trouble imagining how the loss of snowpack and snow molds could help the native perennials better compete against *B. tectorum*.

### Differences in snow mold mortality between greenhouse and field experiments

Why did snow mold reduce seedling survival in the greenhouse experiment but not in the field experiment? A critical difference between the two experiments, besides the lack of actual snow in the greenhouse, was the presence of an intact soil microbial community in the field. We sterilized soils for the greenhouse experiment, allowing the inoculated snow mold to interact with plants in (relative) isolation, whereas in the field experiment snow molds interacted with plants in the presence of the naturally occurring soil microbes. The resident soil microbes could have modified the effect of snow mold.

Another possible reason for the discrepancy between our greenhouse experiments and our field experiment in mortality caused by snow mold is seedling size at time of hardening. A study of *M. nivale* infection of winter wheat seedlings found that the resistance of wheat seedlings is significantly impacted by their size; the smallest seedlings with one or two leaves are less likely than medium-sized plants of two to four leaves to be killed by snow mold (Bruehl and Cunfer 1971). Larger seedlings often senesce tissue that can then be decomposed by snow molds; this tissue senescence is less likely to occur in very small seedlings. In our field experiments, most seedlings were very small when they began to harden; most plants had only one leaf. Greenhouse experiment plants had two leaves and were much larger than field plants.

We also considered the role litter may play in promoting snow mold infection. The greenhouse experiment ensured that mycelia of *M. nivale* were in contact with *B. tectorum* seedlings during the duration of the snowpack simulation. This grain layer effectively functions as soil-top debris. In contrast, grain inoculate was spread more thinly over subplots in the field experiment. Additionally, plots had been cleared of any dead plant material at the onset of the field experiment, possibly reducing the potential for infection by *M. nivale* in the control and snow mold addition subplots. Although *M. nivale* is capable of growing through soil and on soil surfaces (Domsch and Gams 1981), Booth and Taylor (1976) suggested that debris is the primary source of inoculum of *M. nivale*. However, given that snow mold-inoculated subplots did not show a trend of increased mortality, let alone a significant trend, it is unlikely that *M. nivale* is responsible for the overwinter seedling mortality observed in our field experiment and the previous study by Compagnoni and Adler (2014b).

### Does *M. nivale* decrease seed production in *B. tectorum*?

In our field experiment, we found decreased per capita seed production in the plants that survived the winter. Could other fungal pathogens have contributed to this effect? Emergence was uniformly high across treatments, which seems to rule out a role for seed pathogens such as *P. semeniperda*. Infection by head smut might offer a better explanation for the observed effects on seed production. While head smut infection rates showed no evidence of interactive responses to snow melt and fungal treatment, we did see a trend towards increased head smut infection in the snow mold inoculation treatment **[see****Supporting Information—Fig. S2C****]**. It is hard to explain this increase in the early snowmelt treatment, but the potential for interactions between cold and warm season pathogens deserves future attention.

Application of additional fungal cultures and fungicide might have influenced seed production via altered biogeochemical cycling. Schmidt *et al.* (2008) proposed that fast-growing snow molds greatly aid in litter decomposition over the winter and are poised to take advantage of available soil nutrients under cold conditions where much of the microbial activity is suspended. We cannot reject the hypothesis that decomposition of the snow mold culture’s wheat substrate along with mycelial growth into soil may have immobilized nutrient resources that otherwise would have been available to seedlings in the spring. This may have caused stunted growth of plants in snow mold subplots and therefore decreased seed production. However, given that the amount of inoculate was rather low and all other plant litter was cleared in plots at the onset of the experiment, it seems unlikely that this mechanism explains our results. Furthermore, fungicide subplots received the same (sterile) culture but experienced increased seed production under ambient environmental conditions, indicating that the addition of organic matter with our inoculation did not directly deplete nutrient availability for *B. tectorum*.

We speculate that *M. nivale* did infect plants under ambient snowpack conditions, but to a lesser extent than we observed in our greenhouse experiments. Rather than causing outright mortality, a low-grade infection may have depleted some carbohydrate and nutrient reserves and retarded growth (Gaudet *et al.* 1999). Although *M. nivale* is most active under cool, wet conditions (Matsumoto 1994; Hoshino *et al.* 2009), *M. nivale* may be associated with every growth stage of a plant’s lifecycle (Matsumoto 1994).

### Management implications

The utility of our research is in helping land managers anticipate how climate change will alter the trajectory of the *B. tectorum* invasion in the Intermountain West. Our results indicate that climate change will increase population growth rates of *B. tectorum* at sites where the depth and duration of snowpack will decrease in the future, and that release from subnivean pathogens may be one contributing cause. These findings extend results from experiments suggesting that warmer winter conditions may exacerbate impacts of the *B. tectorum* invasion (Chambers *et al.* 2007; Concilio *et al.* 2013; Compagnoni and Adler 2014a) in the higher elevation, cooler and wetter portion of the sagebrush steppe that has historically shown more resistance and resilience to invasion. While a recent model comparison study predicted only modest direct effects of climate change on sagebrush plants at cool, moist sites (Renwick *et al.* 2018), our work suggests the potential for negative indirect effects resulting from interactions between reductions in snowpack caused by climate change, the *B. tectorum* invasion and altered fire regimes. If reduced snowpack results in the loss of a native biological control agent, as our results imply, these important areas of intact sagebrush steppe may become more vulnerable to invasion. Similar interactions between reduced snowpack and the effects of subnivean pathogens should be considered in other temperate ecosystems as well.

## Supporting Information

The following additional information is available in the online version of this article—

**Table S1.** Seedling survival in the growth chamber experiment.

**Table S2.** Likelihood ratio tests for emergence models.

**Table S3.** The statistical model for individual survival selected on the basis of likelihood ratio tests.

**Table S4.** The statistical models for fecundity (seed production), biomass, head smut infection rate and population growth rate, λ, selected on the basis of likelihood ratio tests.

**Table S5.** Likelihood ratio tests for models of individual biomass.

**Figure S1.** Experimental design.

**Figure S2.** *Bromus tectorum* performance in the field experiment.

**Figure S3.** *Bromus tectorum* seed production as a function of above-ground biomass.

plz043_suppl_Supplementary_Material

## Data

All data and computer code necessary to reproduce the analyses are available at the Dryad Digital Repository: doi:10.5061/dryad.v8kj429.

## Sources of Funding

Funding was provided by the National Science Foundation (DEB-1054040 and DEB-1353039), the Utah State University Ecology Center and the Utah Agriculture Experiment Station, Utah State University, which approves this contribution as journal paper number 9084.

## Contributions by the Authors

All authors helped design the experiments and contributed to data analyses; D.M.S. wrote the first draft of the manuscript, and all authors contributed to editing.

## Conflict of Interest

None declared.

## References

[CIT0001] Balch JK, Bradley BA, D’Antonio CM, Gómez-Dans J 2013 Introduced annual grass increases regional fire activity across the arid western USA (1980-2009). Global Change Biology 19:173–183. 23504729 10.1111/gcb.12046

[CIT0002] Bates D, Mächler M, Bolker B, Walker S 2015 Fitting linear mixed-effects models using lme4. Journal of Statistical Software 67:1–48.

[CIT0003] Beckstead J, Meyer SE, Molder CJ, Smith C 2007 A race for survival: can *Bromus tectorum* seeds escape *Pyrenophora semeniperda*-caused mortality by germinating quickly? Annals of Botany 99:907–914. 17353206 10.1093/aob/mcm028 PMC2802916

[CIT0004] Blumenthal DM, Kray JA, Ortmans W, Ziska LH, Pendall E 2016 Cheatgrass is favored by warming but not CO2 enrichment in a semi-arid grassland. Global Change Biology 22:3026–3038. 27090757 10.1111/gcb.13278

[CIT0005] Booth RH, Taylor GS 1976 *Fusarium* diseases of cereals: X. Straw debris as a source of inoculum for infection of wheat by *Fusarium nivale* in the field. Transactions of the British Mycological Society 66:71–75.

[CIT0006] Boyte SP, Wylie BK, Major DJ 2016 Cheatgrass percent cover change: comparing recent estimates to climate change − driven predictions in the northern Great Basin. Rangeland Ecology & Management 69:265–279.

[CIT0007] Bradley BA 2009 Regional analysis of the impacts of climate change on cheatgrass invasion shows potential risk and opportunity. Global Change Biology 15:196–208.

[CIT0008] Bradford JB, Lauenroth WK 2006 Controls over invasion of *Bromus tectorum*: the importance of climate, soil, disturbance and seed availability. Journal of Vegetation Science 17:693–704.

[CIT0009] Bradley BA, Mustard JF 2006 Characterizing the landscape dynamics of an invasive plant and risk of invasion using remote sensing. Ecological Applications 16:1132–1147. 16827008 10.1890/1051-0761(2006)016[1132:ctldoa]2.0.co;2

[CIT0010] Brooks PD, Williams MW 1999 Snowpack controls on nitrogen cycling and export in seasonally snow-covered catchments. Hydrological Processes 13:2177–2190.

[CIT0011] Bruehl GW, Cunfer B 1971 Physiologic and environmental factors that affect the severity of snow mold of wheat. Phytopathology 61:792.

[CIT0012] Bukowski BE, Baker WL 2012 Historical fire regimes, reconstructed from land-survey data, led to complexity and fluctuation in sagebrush landscapes. Ecological Applications 23:546–564. 10.1890/12-0844.1 23734485

[CIT0013] Burke IC 1989 Control of nitrogen mineralization in a sagebrush steppe landscape. Ecology 70:1115–1126.

[CIT0014] Chambers JC, Bradley BA, Brown CS, D’Antonio C, Germino MJ, Grace JB, Hardegree SP, Miller RF, Pyke DA. 2014a Resilience to stress and disturbance, and resistance to *Bromus tectorum* l. invasion in cold desert shrublands of western North America. Ecosystems 17:360–375.

[CIT0015] Chambers JC, Miller RF, Board DI, Pyke DA, Roundy BA, Grace JB, Schupp EW, Tausch RJ. 2014b Resilience and resistance of sagebrush ecosystems: implications for state and transition models and management treatments. Rangeland Ecology & Management 67:440–454.

[CIT0016] Chambers J, Roundy B, Blank R, Meyer S, Whittaker A 2007 What makes Great Basin sagebrush ecosystems invisible by *Bromus tectorum*? Ecological Monographs 77:145.

[CIT0017] Cline DW 1997 Snow surface energy exchanges and snowmelt at a continental, midlatitude Alpine site. Water Resources Research 33:689–701.

[CIT0018] Clow DW 2009 Changes in the timing of snowmelt and streamflow in Colorado: a response to recent warming. Journal of Climate 23:2293–2306.

[CIT0019] Compagnoni A, Adler PB 2014a Warming, soil moisture, and loss of snow increase *Bromus tectorum*’s population growth rate. Elementa: Science of the Anthropocene 2:000020.

[CIT0020] Compagnoni A, Adler PB 2014b Warming, competition, and *Bromus tectorum* population growth across an elevation gradient. Ecosphere 5:art121–art121.

[CIT0021] Concilio AL, Loik ME, Belnap J 2013 Global change effects on *Bromus tectorum* L. (Poaceae) at its high-elevation range margin. Global Change Biology 19:161–172. 23504728 10.1111/gcb.12032

[CIT0022] D’Antonio CM, Vitousek PM 1992 Biological invasions by exotic grasses, the grass fire cycle, and global change. Annual Review of Ecology and Systematics 23:63–87.

[CIT0023] Domsch KH, Gams W 1981 Compendium of soil fungi. London; New York: Academic Press Inc.

[CIT0024] Fournier DA, Skaug HJ, Ancheta J, Ianelli J, Magnusson A, Maunder MN, Nielsen A, Sibert J. 2012 AD Model Builder: using automatic differentiation for statistical inference of highly parameterized complex nonlinear models. *Optimization Methods* and *Software* 27:233–249.

[CIT0025] Gaudet DA, Laroche A, Yoshida M 1999 Low temperature-wheat-fungal interactions: a carbohydrate connection. Physiologia Plantarum 106:437–444.

[CIT0026] Groffman PM, Driscoll CT, Fahey TJ, Hardy JP, Fitzhugh RD, Tierney GL 2001 Colder soils in a warmer world: a snow manipulation study in a northern hardwood forest ecosystem. Biogeochemistry 56:135–150.

[CIT0027] Hanser SE, Manier DJ 2013 Greater Sage-grouse national research strategy. Reston, VA: U.S. Geological Survey.

[CIT0028] Harte J, Shaw R 1995 Shifting dominance within a montane vegetation community: results of a climate-warming experiment. Science 267:876–880. 17813919 10.1126/science.267.5199.876

[CIT0029] Henry HAL 2008 Climate change and soil freezing dynamics: historical trends and projected changes. Climatic Change 87:421–434.

[CIT0030] Hooker TD, Stark JM, Norton U, Leffler AJ, Peek M, Ryel R 2008 Distribution of ecosystem C and N within contrasting vegetation types in a semiarid rangeland in the Great Basin, USA. Biogeochemistry 90:291–308.

[CIT0031] Hoshino T, Xiao N, Tkachenko OB 2009 Cold adaptation in the phytopathogenic fungi causing snow molds. Mycoscience 50:26–38.

[CIT0032] Inouye DW 2008 Effects of climate change on phenology, frost damage, and floral abundance of montane wildflowers. Ecology 89:353–362. 18409425 10.1890/06-2128.1

[CIT0033] Klemmedson JO, Smith JG 1964 Cheatgrass (*Bromus tectorum* L.). The Botanical Review 30:226–262.

[CIT0034] Knick S, Dobkin D, Rotenberry J, Schroeder M, Vander Haegen M, van Riper C III 2003 Teetering on the edge or too late? Conservation and research issues for avifauna of sagebrush habitats. The Condor 105:611–634.

[CIT0035] Knowles N, Dettinger M, Cayan D 2006 Trends in snowfall versus rainfall in the Western United States. Journal of Climate 19:4545–4559.

[CIT0036] Kreyling J 2010 Winter climate change: a critical factor for temperate vegetation performance. Ecology 91:1939–1948. 20715613 10.1890/09-1160.1

[CIT0037] Leifeld P 2013 texreg: conversion of statistical model output in R to \LaTeX and HTML tables. Journal of Statistical Software 55:1–24.

[CIT0038] Luck J, Spackman M, Freeman A, Tre˛bicki P, Griffiths W, Finlay K, Chakraborty S 2011 Climate change and diseases of food crops. Plant Pathology 60:113–121.

[CIT0039] Mack RN 1981 Invasion of *Bromus tectorum* L. into western North America: an ecological chronicle. Agro-Ecosystems 7:145–165.

[CIT0040] Mastin MC, Chase KJ, Dudley RW 2010 Changes in spring snowpack for selected basins in the United States for different climate-change scenarios. Earth Interactions 15:1–18.

[CIT0041] Matsumoto N 1994 Ecological adaptations of low temperature plant pathogenic fungi to diverse winter climates. Canadian Journal of Plant Pathology 16:237–240.

[CIT0042] Meyer SE, Beckstead J, Allen PS, Smith DC 2008 A seed bank pathogen causes seedborne disease: *Pyrenophora semeniperda* on undispersed grass seeds in western North America. Canadian Journal of Plant Pathology 30:525–533.

[CIT0043] Meyer SE, Beckstead J, Pearce J 2016 Community ecology of fungal pathogens on *Bromus tectorum.* In: Germino MJ, Chambers JC, Brown CS, eds. Exotic Brome-grasses in arid and semiarid ecosystems of the Western US: causes, consequences, and management implications. Cham: Springer Series on Environmental Management, Springer International Publishing, 193–223.

[CIT0044] Meyer SE, Franke J-L, Baughman OW, Beckstead J, Geary B 2014 Does *Fusarium*-caused seed mortality contribute to *Bromus tectorum* stand failure in the Great Basin? Weed Research 54:511–519.

[CIT0045] Meyer SE, Quinney D, Nelson DL, Weaver J 2007 Impact of the pathogen *Pyrenophora semeniperda* on *Bromus tectorum* seedbank dynamics in North American cold deserts. Weed Research 47:54–62.

[CIT0046] Miller RF, Wigand PE 1994 Holocene changes in semiarid Pinyon-Juniper woodlands. BioScience 44:465–474.

[CIT0047] Modjo HS, Hendrix JW 1986 The mycorrhizal fungus *Glomus macrocarpum* as a cause of tobacco stunt disease. Phytopathology 76:688.

[CIT0048] Monson RK, Lipson DL, Burns SP, Turnipseed AA, Delany AC, Williams MW, Schmidt SK 2006 Winter forest soil respiration controlled by climate and microbial community composition. Nature 439:711–714. 16467835 10.1038/nature04555

[CIT0049] Mordecai EA 2013 Despite spillover, a shared pathogen promotes native plant persistence in a cheatgrass-invaded grassland. Ecology 94:2744–2753. 24597221 10.1890/13-0086.1

[CIT0050] Nakajima T, Abe J 1994 Development of resistance to *Microdochium nivale* in winter wheat during autumn and decline of the resistance under snow. Canadian Journal of Botany 72:1211–1215.

[CIT0051] Pautasso M, Döring TF, Garbelotto M, Pellis L, Jeger MJ 2012 Impacts of climate change on plant diseases—opinions and trends. European Journal of Plant Pathology 133:295–313.

[CIT0052] Prevéy JS, Seastedt TR 2015 Increased winter precipitation benefits the native plant pathogen *Ustilago bullata* that infects an invasive grass. Biological Invasions 17:3041–3047.

[CIT0053] R Development Core Team 2017 R: a language and environment for statistical computing. R Foundation for Statistical Computing, Vienna, Austria.

[CIT0054] Renwick KM, Curtis C, Kleinhesselink AR, Schlaepfer D, Bradley BA, Aldridge CL, Poulter B, Adler PB 2018 Multi-model comparison highlights consistency in predicted effect of warming on a semi-arid shrub. Global Change Biology 24:424–438. 28895271 10.1111/gcb.13900

[CIT0055] Schlaepfer DR, Lauenroth WK, Bradford JB 2012a Consequences of declining snow accumulation for water balance of mid‐latitude dry regions. Global Change Biology 18:1988–1997.

[CIT0056] Schlaepfer DR, Lauenroth WK, Bradford JB 2012b Effects of ecohydrological variables on current and future ranges, local suitability patterns, and model accuracy in big sagebrush. Ecography 35:374–384.

[CIT0057] Schmidt SK, Wilson KL, Meyer AF, Gebauer MM, King AJ 2008 Phylogeny and ecophysiology of opportunistic “snow molds” from a subalpine forest ecosystem. Microbial Ecology 56:681–687. 18443847 10.1007/s00248-008-9387-6

[CIT0058] Smith DC, Meyer SE, Anderson VJ 2008 Factors affecting *Bromus tectorum* seed bank carryover in western Utah. Rangeland Ecology and Management 61:430–436.

[CIT0059] Sprague R 1953 Root and crown rots of the grasses. In: May C, Brierley P, Clayton EE, Dunegan JC, Kreitlow KW, McClellan WD, Miller PR, Rodenhiser HA, Zaumeyer WJ, eds. Plant diseases: the yearbook of agriculture 1953. Washington, DC: USDA, 267–272.

[CIT0060] Stewart IT 2009 Changes in snowpack and snowmelt runoff for key mountain regions. Hydrological Processes 23:78–94.

[CIT0061] Stewart G, Hull AC 1949 Cheatgrass (*Bromus tectorum* l.)--an ecologic intruder in southern Idaho. Ecology 30:58–74.

[CIT0062] Tisdale EW, McLean SE 1954 Range resources and their management in British Columbia. Journal of Range Management 7:3–9.

[CIT0063] Tylianakis JM, Didham RK, Bascompte J, Wardle DA 2008 Global change and species interactions in terrestrial ecosystems. Ecology Letters 11:1351–1363. 19062363 10.1111/j.1461-0248.2008.01250.x

[CIT0064] van Auken OW 2000 Shrub invasions of North American semiarid grasslands. Annual Review of Ecology & Systematics 31:197–215.

[CIT0065] van der Putten WH, Macel M, Visser ME 2010 Predicting species distribution and abundance responses to climate change: why it is essential to include biotic interactions across trophic levels. Philosophical Transactions of the Royal Society of London. Series B, Biological Sciences 365:2025–2034. 20513711 10.1098/rstb.2010.0037 PMC2880132

[CIT0066] Wahren C-HA, Walker MD, Bret-Harte MS 2005 Vegetation responses in Alaskan arctic tundra after 8 years of a summer warming and winter snow manipulation experiment. Global Change Biology 11:537–552.

[CIT0067] Wipf S, Rixen C, Mulder CPH 2006 Advanced snowmelt causes shift towards positive neighbour interactions in a subarctic tundra community. Global Change Biology 12:1496–1506.

