# Supplementary material for: Climate change, snow mold and the Bromus tectorum invasion: mixed evidence for release from cold weather pathogens
Source: AoB Plants. 2019 Jul 16;11(5):plz043. doi: 10.1093/aobpla/plz043 (PMC6756605; doi:10.1093/aobpla/plz043)
Supplement: plz043_suppl_Supplementary_Material [file plz043_suppl_supplementary_material.pdf]

Supporting Information

Climate change, snow mold, and the *Bromus tectorum* invasion: release from cold weather pathogens?

Danielle M. Smull, Nicole Pendleton, Andrew R. Kleinhesselink, and Peter B. Adler

Supplementary Tables

Table S1. Seedling survival in the growth chamber experiment.

	Coefficient
(Intercept)	0.15 (0.18)
+Snow mold	-2.98 (0.43)***
Log Likelihood	-110.57
Deviance	221.14
Num. obs.	247

$^{***}p < 0.001$, $^{**}p < 0.01$, $^{*}p < 0.05$

Table S2. Likelihood ratio tests for emergence models. Each row, except for the first, compares the listed model with the simpler model in the previous row.

Model	AIC	logLik	deviance	χ^2	df	p-value
NULL	3693.15	-1843.57	3687.15			
snowmelt	3695.01	-1843.51	3687.01	0.13	1	0.7147
snowmelt + fungal	3698.10	-1843.05	3686.10	0.91	2	0.6329
snowmelt×fungal	3700.78	-1842.39	3684.78	1.32	2	0.5173

Table S3. The statistical model for individual survival selected on the basis of likelihood ratio tests.

	**Survival**
(Intercept)	-0.35 (0.26)
Snow melt	1.39 (0.33)***
Fungicide	0.52 (0.21)*
Snow mold	0.07 (0.21)
AIC	3241.52
BIC	3277.01
Log Likelihood	-1614.76
Num. obs.	2741
Num. groups: Subplot.ID:Plot.ID	36
Num. groups: Plot.ID	12
Var: Subplot.ID:Plot.ID (Intercept)	0.20
Var: Plot.ID (Intercept)	0.24

$^{***}p < 0.001$, $^{**}p < 0.01$, $^{*}p < 0.05$

Table S4. The statistical models for fecundity (seed production), biomass, head smut infection rate, and population growth rate, λ, selected on the basis of likelihood ratio tests.

	**Fecundity**	**Biomass**	**Head smut**	**log λ**
(Intercept)	0.4409 (0.2980)	0.0252 (0.0093)**	0.2185 (0.2195)	-0.8635 (0.3022)**
Snow melt	0.4495 (0.3900)	0.0084 (0.0132)	0.2035 (0.2391)	1.1719 (0.3721)**
Fungicide	0.4094 (0.2359)	0.0055 (0.0053)	-0.2880 (0.1886)	0.3357 (0.2575)
Snow mold	-0.5810 (0.3064)	-0.0049 (0.0053)	0.2670 (0.1704)	-0.4420 (0.2575)
Snow melt x Fungicide	-0.3835 (0.2782)	-0.0078 (0.0075)		
Snow melt x Snow mold	0.5562 (0.3428)	0.0084 (0.0075)		
Variance: Plot.ID	0.3057		0.0957	
Dispersion: parameter	12.4210		14.2900	
Dispersion: SD	3.7229		4.2007	
AIC	388.8920	-141.5440	360.7440	94.6846
BIC	401.5602	-128.8759	370.2451	104.1857
Log Likelihood	-186.4460	78.7720	-174.3720	-41.3423
Num. obs.	36	36	36	36
Num. groups: Plot.ID	12	12	12	12
Var: Plot.ID (Intercept)		0.0004		0.2828
Var: Residual		0.0001		0.3978

$^{***}p < 0.001$, $^{**}p < 0.01$, $^{*}p < 0.05$

Table S5. Likelihood ratio tests for models of individual biomass.

Model	AIC	logLik	deviance	χ^2	df	p-value
NULL	-196.54	101.27	-202.54			
snowmelt	-195.09	101.55	-203.09	0.55	1	0.4581
snowmelt + fungal	-191.48	101.74	-203.48	0.39	2	0.8238
snowmelt×fungal	-192.52	104.26	-208.52	5.04	2	0.0805

Supplementary Figures

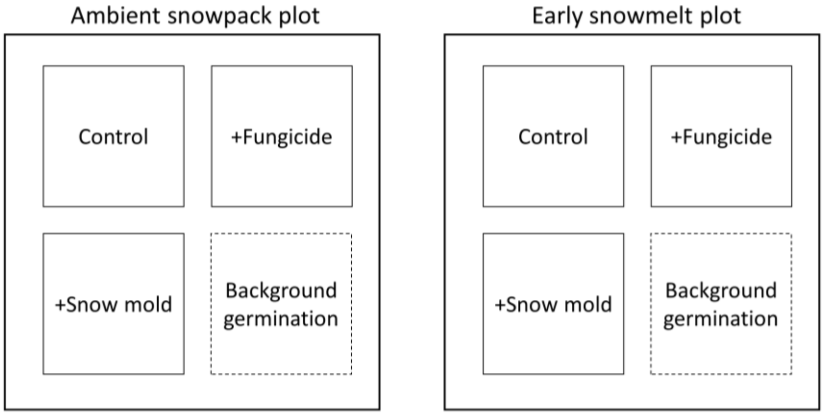

Fig. S1. Experimental design. The snowmelt treatment was applied at the whole plot level: half the plots were ambient snowpack controls, half were placed under infrared heaters to melt snow from January through March. The fungicide treatments were applied at the subplot level. Snow mold subplots were inoculated with a wheat seed culture. Fungicide and controls plots received sterile wheat seed. *B. tectorum* was seeded in all three treated subplots. The fourth subplot was an unseeded control to monitor background germination of *B. tectorum*.

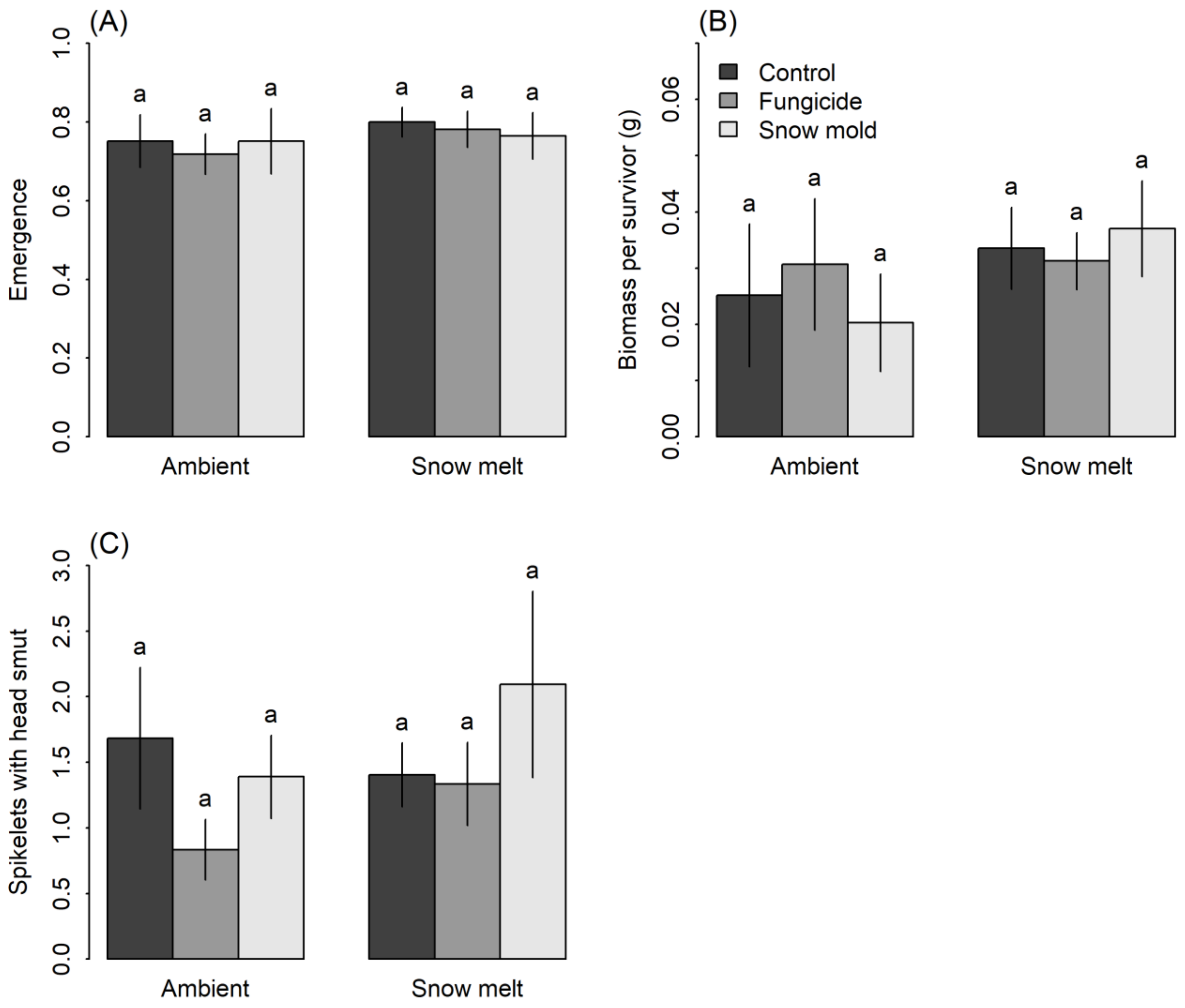

Figure S2. *B. tectorum* performance in the field experiment. (A) Emergence, (B) Per capita aboveground biomass production of *Bromus tectorum* at time of maturity and (C) spikelets infected with head smut per surviving plant. Values are treatment means, and error bars indicate ± 1 standard error. Letters indicate statistically significant differences between treatments based on custom contrasts. Note that head smut means shown here (C) are spikelets with head smut per subplot / survivors, whereas our statistical analysis, and associated contrasts, modeled spikelets with head smut on the log scale with a log survivors offset.

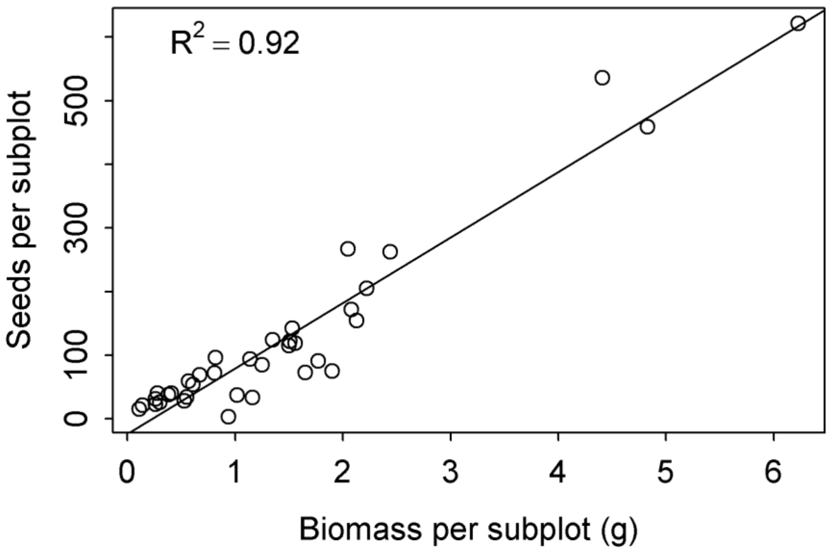

Figure S3. *B. tectorum* seed production as a function of aboveground biomass. Points represent individual subplot biomass and seed production values.
